# Laparoscopic radical antegrade modular pancreatosplenectomy vesus laparoscopic distal pancreatosplenectomy for left-sided pancreatic cancer: a systematic review and meta-analysis

**DOI:** 10.3389/fonc.2025.1510342

**Published:** 2025-02-14

**Authors:** Xutao Jiang, Yu Zhu, Jianwei Li, Wei Li, Weizong Zheng, Caiming Xu, Guixin Zhang

**Affiliations:** ^1^ Department of General Surgery, the Second Hospital of Dalian Medical University, Dalian, China; ^2^ Department of General Surgery, Dongxiang District People’s Hospital, Fuzhou, China; ^3^ Department of Intensive Care Medicine, Dongxiang District People’s Hospital, Fuzhou, China; ^4^ Department of Molecular Diagnostics and Experimental Therapeutics, Beckman Research Institute of City of Hope, Biomedical Research Center, Comprehensive Cancer Center, Monrovia, CA, United States; ^5^ Department of General Surgery, The First Affiliated Hospital of Dalian Medical University, Dalian, China; ^6^ Institute (College) of Integrative Medicine, Dalian Medical University, Dalian, Liaoning, China

**Keywords:** laparoscopic, radical antegrade modular pancreatosplenectomy, pancreatic cancer, distal pancreatosplenectomy, meta-analysis

## Abstract

**Objective:**

We aimed to compare the perioperative outcomes and postoperative complications of laparoscopic radical antegrade modular pancreatosplenectomy (L-RAMPS) versus laparoscopic distal pancreatosplenectomy (L-DPS) for left-sided pancreatic cancer through a meta-analysis.

**Methods:**

A systematic review and meta-analysis, conducted in accordance with the Preferred Reporting Items for Systematic Reviews and Meta-Analyses (PRISMA) guidelines, were performed. Literature searches were conducted in PubMed, Web of Science, Cochrane Library, and Embase for studies published from their inception up to June 14th, 2024.

**Results:**

A total of three retrospective studies involving 242 patients were included in this meta-analysis, with 116 patients in the L-RAMPS group and 126 in the L-DPS group. The meta-analysis results indicated that L-RAMPS was associated with the retrieval of more lymph nodes (MD: 3.06; 95% CI: 2.51 to 3.62, p < 0.00001) and longer operative time (MD: 20.05; 95% CI: 13.97 to 26.12, p < 0.00001) compared to L-DPS for left-sided pancreatic cancer patients. However, no significant differences were observed between the two groups in terms of R0 resection margins, the incidence of pancreatic fistula (Grade B and C), postpancreatectomy hemorrhage, or postoperative complications (Clavien-Dindo Grades II and III).

**Conclusions:**

In patients with left-sided pancreatic cancer, L-RAMPS resulted in the retrieval of more lymph nodes, a longer operative time, and a similar incidence of postoperative complications compared to L-DPS. Larger sample sizes, extended follow-up periods, and well-conducted randomized controlled trials are needed to further validate these findings

**Systematic review registration:**

https://www.crd.york.ac.uk/PROSPERO/display_record.php?RecordID=558977, identifier CRD42024558977.

## 1 Introduction

Pancreatic ductal adenocarcinoma (PDAC) is a highly aggressive tumor with increasing global incidence, making its early diagnosis and management particularly challenging due to its rapid progression (1). Typically, it is diagnosed during an advanced stage of the disease when the tumor has spread beyond the margins of the pancreas to adjacent tissues or distant organs. (2)

Surgical resection remains the only potentially curative treatment for PDAC (3, 4). Specifically, laparoscopic distal pancreatosplenectomy (L-DPS) is recommended as the standard procedure for left-sided PDAC resection (5). A number of studies have demonstrated that L-DPS is a feasible, safe, and oncologically equivalent treatment for PDAC (6, 7). However, uncertainties remain regarding the extent of posterior resection and the effectiveness of achieving complete lymph node resection during L-DPS (7).

A radical antegrade modular pancreatosplenectomy (RAMPS) procedure was developed in 2003 by Strasberg in order to achieve a radical operation with the most extensive lymphadenectomy possible (8). In comparison with DPS, RAMPS attempts to achieve negative retroperitoneal margins and higher lymph node retrieval in order to improve survival outcomes (9–11), Despite this, comparisons between RAMPS and DPS have yielded mixed results, with recent meta-analyses suggesting minimal impact on prognosis from RAMPS for left-sided pancreatic cancer (11–15).

Given the importance of understanding the comparative effectiveness and safety of these surgical techniques, and the limited clinical analysis of laparoscopic RAMPS (L-RAMPS) versus L-DPS, this meta-analysis aims to evaluate the perioperative outcomes and postoperative complications associated with each approach in patients with left-sided pancreatic cancer.

## 2 Methods

This study adhered to the Cochrane Handbook for Systematic Reviews of Interventions, Preferred Reporting Items for Systematic Reviews and Meta-analyses (PRISMA) reporting guidelines, and AMSTAR 2 guidelines (assessing the methodological quality of systematic reviews) (16–18). The current meta-analysis was registered on the PROSPERO website (registration number: CRD42024558977). As this research involved a secondary analysis of existing published data, ethical approval was not necessary.

### 2.1 Database search

Literature searching was conducted in PubMed, Web of Science, Cochrane Library and Embase for studies published rom their inception through June 14th, 2024. The search had no language or regional restrictions. Keywords and medical subject heading (MeSH) terms used included “laparoscopic”, “radical antegrade modular pancreatosplenectomy”, “distal pancreatosplenectomy”, and “pancreatic cancer”. Additionally, we examined the bibliographies of relevant articles to identify further studies.

### 2.2 Eligibility criteria

Inclusion criteria were as follows: (1) clinical studies comparing L-RAMPS and L-DPS for patients with left-sided PDAC; (2) full-text articles reporting at least one outcome of interest, such as perioperative outcomes or postoperative complications; and (3) in the case of duplicate reports, the study with the most comprehensive, up-to-date, and largest dataset was included.

Exclusion criteria included: (1) duplicate publications; (2) studies lacking complete and valid outcome data for statistical analysis; and (3) case reports, reviews, animal studies, editorial comments, meeting abstracts, and other unrelated research.

### 2.3 Data extraction

Two investigators independently screened and evaluated the studies based on the inclusion criteria and extracted relevant data from the included studies. In the case of disputes, a third investigator was consulted to resolve them. The following data were extracted: (1) baseline data: name of the first author, year of publication, country, study design, sample size, patients’ age, body mass index, and tumor size; (2) perioperative outcomes: operative time, estimated blood loss, R0 transection margin, and retrieved lymph nodes; and (3) postoperative complications such as pancreatic fistula (Grade B and C), Clavien - Dindo classification (Grade 2 and 3), postpancreatectomy hemorrhage, postoperative mortality, and long-term outcomes.

### 2.4 Quality assessment

The quality of the retrospective studies was assessed using the Methodological Index for Non-Randomized Studies (MINORS), which includes 12 evaluation items. Each item was scored on a scale of 0 to 2 points: 0 points indicated the item was not reported, 1 point indicated the item was reported with insufficient information, and 2 points indicated the item was reported with sufficient information (19). The assessment was independently conducted by two authors, with any disagreements resolved through discussion with a third author.

### 2.5 Statistical analysis

Review Manager Version 5.4 (The Cochrane Collaboration, Oxford, UK) was used for the statistical analysis. Continuous variables were expressed as weighted mean differences (WMD) and 95% confidence interval (CI), and binary variables were represented by odds ratios (OR) and 95% CI. When only median and extreme values were reported in the study, the mean and standard deviation (SD) were calculated from the median and range, as described by Hozo et al. (20) Heterogeneity among the studies was assessed using the I^2^ statistic (0-50%, low heterogeneity; 50-75%, moderate heterogeneity; and ≥75%, high heterogeneity) (21). For studies with obvious heterogeneity and an I^2^-value of more than 50%, the random-effects model was adopted. Sensitivity analyses were performed as appropriate. Publication bias was evaluated using Begg’s test and Egger’s test using the Stata software (Stata version 16.0, College Station, Texas, USA). For all tests, a P-value <0.05 (two-sided) was considered to indicate a significant difference.

## 3 Results

**Figure 1:**
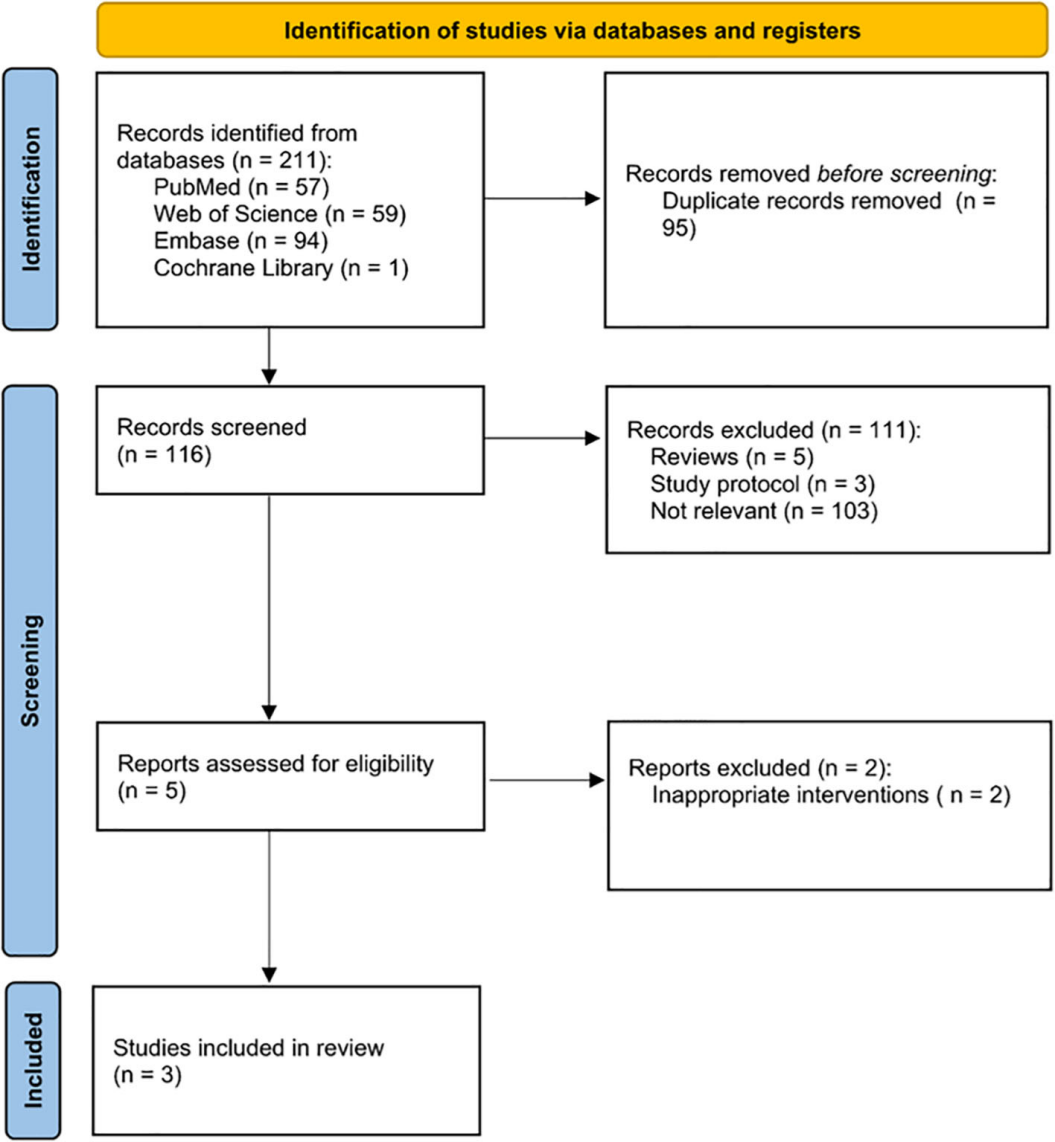
Flow diagram of the study selection process.

A flow diagram of the study selection process can be seen in **Figure 1**. No randomized controlled trials (RCTs) were available for analysis, however, three retrospective comparative studies were eligible (22–24). A total of three retrospective studies with 242 patients, of whom 116 patients were in the L-RAMPS group and 126 in the L-DPS group, were involved in this meta-analysis. The baseline characteristics of these studies, including author, country, study design, sample size, sex, patients’ age, body mass index, tumor size, target outcomes, and MINORS score, are provided in **Table 1**.

**Table 1:** Characteristic of the included studies.

Authors	Year	Country	Study design	Sample size (Female)		Age, years		Body mass index, kg/m^2^		Tumor size, cm		Target outcomes	MINORS score
				L-DPS	L-RAMPS	L-DPS	L-RAMPS	L-DPS	L-RAMPS	L-DPS	L-RAMPS		
Niu et al. (24)	2022	China	Retrospective	59 (22)	50 (19)	66 [38,88]	67 [44,87]	22.4 [14.8,30.5]	22.3 [17.6,30.1]	3.5 [1.5,7.0]	3.5 [1.8,10]	1, 2, 3, 4, 5, 6	19
Li et al. (23)	2024	China	Retrospective	54 (26)	54 (23)	67 [58,71]	67 [59,72]	23.3 [21.8,25.7]	23.3 [21.4,25.0]	3.0 [2.3,3.6]	3.0 [2.2,3.6]	1, 2, 3, 4, 5, 6, 7	19
Borys et al. (22)	2024	Poland	Retrospective	13 (7)	12 (10)	66 [59,88]	70 [66,75]	26.3 [24.8,28.3]	24.4 [22.3,26.1]	4.5 [3.5,5.0]	2.7 [2.1,4.4]	1, 2, 3, 5, 7	17

L-RAMPS: laparoscopic radical antegrade modular pancreatosplenectomy; L-DPS: laparoscopic distal pancreatosplenectomy; MINORS: methodological index for non-randomized studies; Target outcomes: 1.operative time; 2.estimated blood loss; 3.R0 transection margin; 4.retrieved lymph nodes; 5.pancreatic fistula (Grade B and C); 6.Clavien - Dindo classification (Grade II and III); 7.postpancreatectomy hemorrhage. Continuous data are presented as medians with first and third quartiles (Q1-Q3).

### 3.1 Perioperative outcomes

#### 3.1.1 Operative time

**Figure 2:**
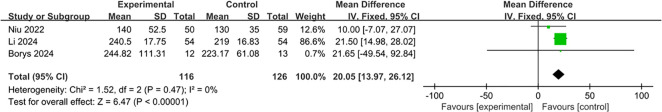
Forest plot of operative time.

All of the three studies were included in the analysis of the operative time (22–24). The operative time was significantly shorter in the L-DPS group than that in the L-RAMPS group (MD: 20.05; 95% CI: 13.97 to 26.12, p < 0.00001). The between-study heterogeneity was low (I^2^ = 0%, p = 0.47) (**Figure 2**).

#### 3.1.2 Estimated blood loss

**Figure 3:**
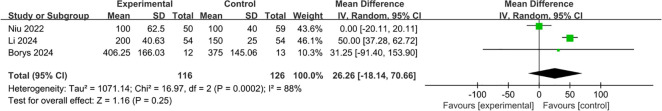
Forest plot of estimated blood loss.

All three studies were included in the analysis of estimated blood loss (22–24). No significant difference in estimated blood loss was observed between the L-RAMPS group and the L-DPS group (MD: 26.26; 95% CI: -18.14 to 70.66, p = 0.25). The between-study heterogeneity was significant (I^2^ = 88%, p = 0.0002) (**Figure 3**).

#### 3.1.3 Retrieved lymph nodes

**Figure 4:**
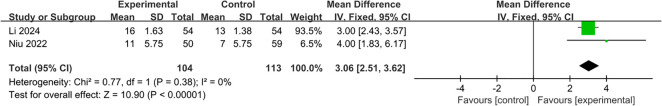
Forest plot of number of retrieved lymph nodes.

Two studies were included in the analysis of the number of retrieved lymph node (23, 24). Our analysis demonstrated that the number of retrieved lymph node was less in L-DPS group (MD: 3.06; 95% CI: 2.51 to 3.62, p < 0.00001). Between-study heterogeneity was low (I^2^ = 0%, p = 0.38) (**Figure 4**).

#### 3.1.4 R0 transection margin

**Figure 5:**
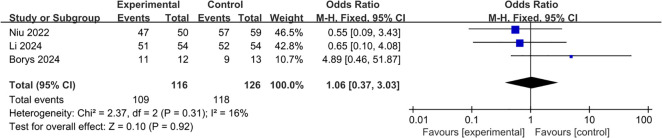
Forest plot of R0 transection margin.

All of the three studies were included in the analysis of R0 transection margin (22–24). Pooling of the results indicated that the L-RAMPS procedure could not decrease the incidence of R0 transection margin compared with the L-DPS procedure (OR: 1.06; 95%CI: 0.37 to 3.03; p = 0.92), and low heterogeneity existed among the included studies (p = 0.31, I^2^ = 16%) (**Figure 5**).

### 3.2 Postoperative outcomes

#### 3.2.1 Pancreatic fistula (Grade B and C)

**Figure 6:**
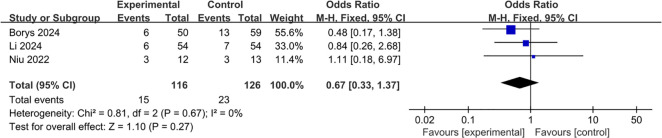
Forest plot of pancreatic fistula (Grade B and C).

All of the three studies were included in the analysis of pancreatic fistula. Pooling of the results indicated that the L-RAMPS procedure could not decrease the incidence of pancreatic fistula compared with the L-DPS procedure (OR: 0.67, 95%CI: 0.33 to 1.37, p = 0.27), and low heterogeneity existed among the included studies (p = 0.67, I^2^ = 0%) (**Figure 6**).

#### 3.2.2 Postpancreatectomy hemorrhage

**Figure 7:**
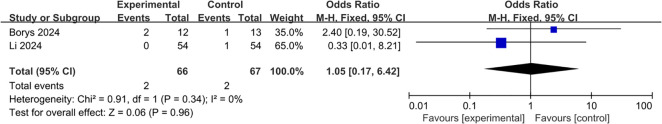
Forest plot of postpancreatectomy hemorrhage.

Two studies were included in the analysis of postpancreatectomy hemorrhage (22, 23). Pooling of the results indicated that the L-RAMPS procedure could not decrease the incidence of postpancreatectomy hemorrhage compared with the L-DPS procedure (OR: 1.05, 95%CI: 0.17 to 6.42, p = 0.96), and low heterogeneity existed among the included studies (p = 0.34, I^2^ = 0%) (**Figure 7**).

#### 3.2.3 Postoperative complications (Clavien-Dindo Grade II and III)

**Figure 8:**
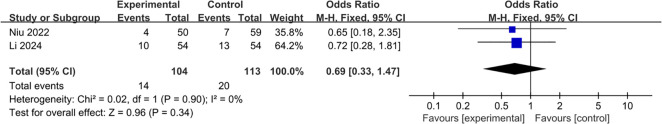
Forest plot of postoperative complications (Clavien-Dindo Grade II and III).

Two studies were included in the analysis of postoperative complications (23, 24). Pooling of the results indicated that the L-RAMPS procedure could not decrease the incidence of postoperative complications (OR: 0.69, 95%CI: 0.33 to 1.47, p = 0.34), and low heterogeneity existed among the included studies (p = 0.90, I^2^ = 0%) (**Figure 8**).

#### 3.2.4 Postoperative mortality

Postoperative mortality related indicators, such as 30-day mortality and 90-day mortality, were only reported in one study (22). L-DPS group has lower 30-day mortality (0 vs. 8.3%, p = N/A) and 90-day mortality (7.7% vs. 16.7%, p = 0.49) than L-RAMPS group, however the difference between the two groups was not statistically significant.

### 3.3 Long−term prognosis

Recurrence-free survival (RFS) was reported in one of the included studies (23). There was no significant difference in RFS observed between these two groups. The 6-month, 1-year, and 2-year RFS rates were 88.9%, 75.1%, and 41.6% for the L-RAMPS group, and 94.4%, 73.7%, and 48.6% for the LDP group, respectively (p = 0.715). Another study compared the disease-free survival (DFS) and overall survival (OS) of two groups (24). According to the univariate analysis, L-RAMPS is not associated with an improvement in either DFS (p = 0.544) or OS (p = 0.336) over L-DPS.

For postoperative recurrence and metastasis, only one included study reported related data (23). The rates of early recurrence were 61.9% in the L-RAMPS group and 66.7% in the L-DPS group (p = 0.757). In the L-RAMPS group, the proportions of local recurrence, liver metastasis, and other distant metastasis were 23.8%, 52.4%, and 23.8%, respectively. In the L-DPS group, the corresponding proportions were 38.9%, 27.8%, and 33.3% (p = 0.293).

### 3.4 Sensitivity analysis and publication bias

**Figure 9:**
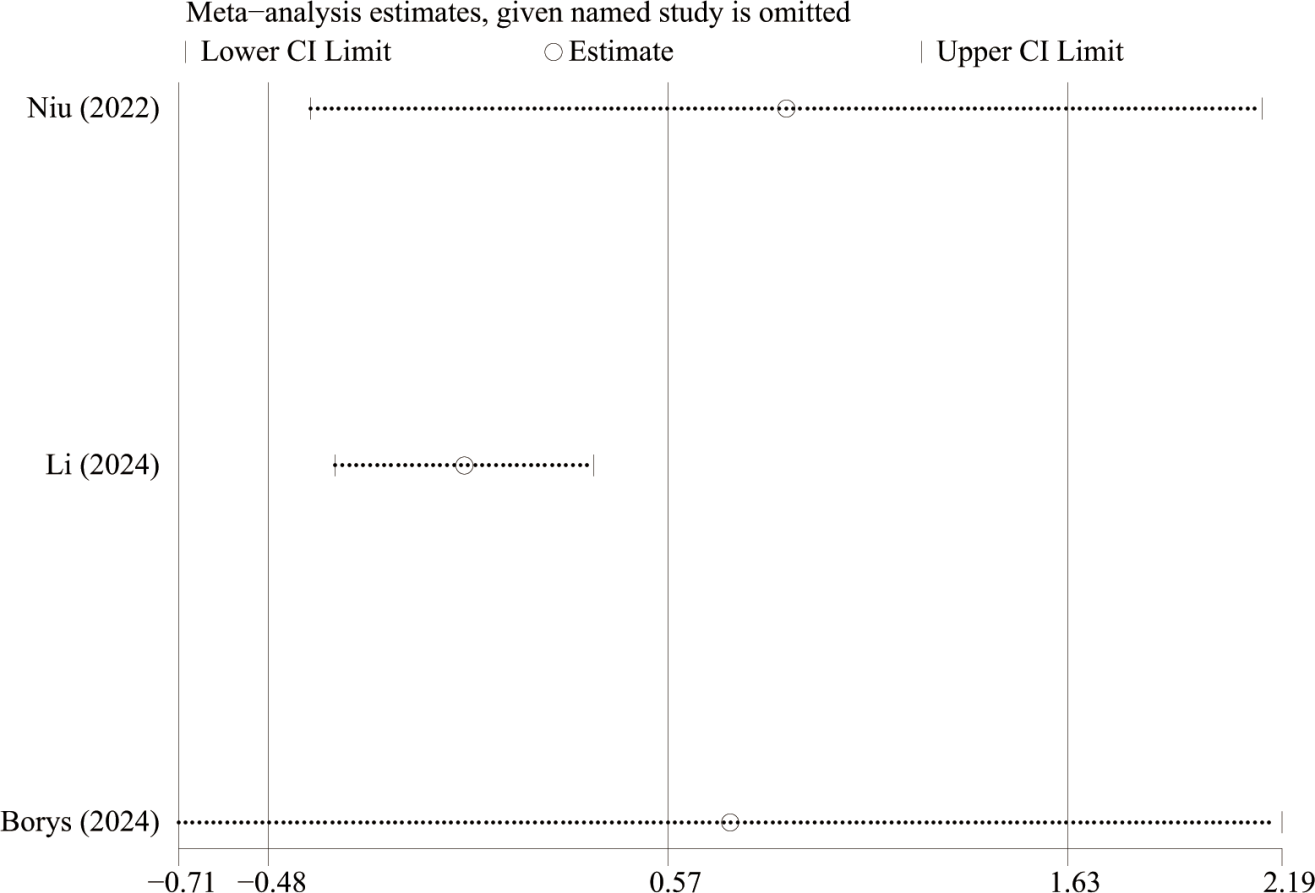
Sensitivity analysis.

We performed sensitivity analysis (**Figure 9**) to estimate the stability of the results via sequentially excluding the results of each individual study. Sensitivity analyses showed that no single article had a strong influence on the results of estimated blood loss. The Egger’s test result was p = 0.055 and the Begg’s test result was p = 0.296 suggesting that there was less possibility of publication bias in this study.

## 4 Discussion

In general, radical surgery plays a crucial role in the treatment of left-sided pancreatic cancer. RAMPS is an advanced surgical procedure designed to achieve complete dissection of D1 lymph nodes and increase the R0 resection rate, both of which are key prognostic factors for patients with left-sided PDAC (9, 25–27).

However, L-RAMPS is infrequently performed due to its technical complexity and the absence of clear superiority over other methods (28). Meta-analyses comparing open RAMPS with open DPS show that while RAMPS may improve disease-free survival (DFS), it has minimal impact on overall survival (OS) and recurrence-free survival (RFS). Although open RAMPS can retrieve more lymph nodes, it does not necessarily enhance R0 resection rates (13). Furthermore, another meta-analysis indicates that L-RAMPS yields similar outcomes to open RAMPS, with the added benefits of minimal invasiveness, such as less blood loss and shorter time to oral feeding. However, L-RAMPS harvested significantly fewer lymph nodes, which may potentially negatively influence the long-term survival of patients with left-sided pancreatic cancer (29).

There remains significant controversy regarding the efficacy and safety of RAMPS and DPS in the treatment of left-sided pancreatic cancer. This meta-analysis offers a comprehensive review of L-DPS and L-RAMPS outcomes in these patients. It represents the first analysis comparing the perioperative and postoperative results of L-RAMPS with L-DPS. Our findings highlight L-RAMPS’s advantage in retrieving more lymph nodes, consistent with previous meta-analyses comparing open-RAMPS and DPS, despite the increased operative time associated with L-RAMPS (13).

Both L-RAMPS and L-DPS demonstrated similar rates of estimated blood loss, R0 resection margins, and postoperative complications. RAMPS employs a no-touch isolation approach to control major blood vessels by separating the pancreatic neck early, which theoretically reduces blood loss (30). Despite this, our meta-analysis found no significant difference in blood loss between the two techniques. Similarly, R0 resection rates were comparable for both methods, though achieving R0 resection is crucial for improving survival in pancreatic cancer (31, 32). The postoperative mortality, including both 30-day and 90-day mortality rates, appears to be similar between the L-RAMPS and L-DPS groups, indicating that L-RAMPS is a safe surgical approach for patients with left-sided pancreatic cancer. Moreover, long−term prognosis, including recurrence-free survival (RFS), disease-free survival (DFS), and overall survival (OS), does not appear to be significantly affected by these two surgical strategies. Although L-RAMPS is technically more complex and raises concerns about postoperative complications, this analysis found no significant differences in rates of pancreatic fistula, postpancreatectomy hemorrhage, post-operative mortality, or other complications between L-RAMPS and L-DPS. The lack of substantial differences suggests that both techniques offer similar efficacy and safety in the treatment of left-sided pancreatic cancer. Therefore, the choice of treatment modality should depend on the surgeon’s expertise, the availability of equipment, and the patient’s expectations, understanding, and cooperation.

This meta-analysis has several limitations. Firstly, it is based on retrospective studies with small sample sizes and a limited number of included studies, which could be influenced by publication bias. Secondly, only retrospective studies included in this meta-analysis, high-quality, large-scale randomized controlled trials are needed to draw definitive conclusions about various outcomes. Thirdly, while the analysis focused on short-term perioperative and postoperative outcomes, long-term survival benefits of L-RAMPS versus L-DPS remain unclear due to limited data. Therefore, further research is needed to explore long-term outcomes, and clinicians should interpret these findings with caution.

## 5 Conclusion

In conclusion, this meta-analysis is the first to demonstrate that L-RAMPS results in a higher number of lymph nodes retrieved and a longer operative time compared to L-DPS for left-sided pancreatic cancer. However, both approaches yield similar outcomes in terms of R0 resection margins and postoperative complications. To further validate these findings, larger sample sizes, extended follow-up periods, and well-conducted randomized controlled trials are necessary.

## Data Availability

The original contributions presented in the study are included in the article/supplementary material. Further inquiries can be directed to the corresponding author.
