# Erratum: Laparoscopic radical antegrade modular pancreatosplenectomy vesus laparoscopic distal pancreatosplenectomy for left-sided pancreatic cancer: a systematic review and meta-analysis

**DOI:** 10.3389/fonc.2025.1601978

**Published:** 2025-04-11

**Authors:** Frontiers Production Office

**Affiliations:** Frontiers Media SA, Lausanne, Switzerland

**Keywords:** laparoscopic, radical antegrade modular pancreatosplenectomy, pancreatic cancer, distal pancreatosplenectomy, meta-analysis

Due to a production error, the fundings were erroneously added to the online version of the article, which has been removed now.

The publisher apologizes for this mistake.

The original version of this article has been updated.

